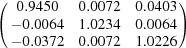# Crystallographic features of the martensitic transformation and their impact on variant organization in the intermetallic compound Ni_50_Mn_38_Sb_12_ studied by SEM/EBSD

**DOI:** 10.1107/S2052252517011332

**Published:** 2017-09-01

**Authors:** Chunyang Zhang, Yudong Zhang, Claude Esling, Xiang Zhao, Liang Zuo

**Affiliations:** a Key Laboratory for Anisotropy and Texture of Materials (Ministry of Education), Northeastern University, Shenyang 110819, People’s Republic of China; b Laboratoire d’Étude des Microstructures et de Mécanique des Matériaux (LEM3), CNRS UMR 7239, Université de Lorraine, Metz 57073, France; c Laboratory of Excellence on Design of Alloy Metals for low-mAss Structures (DAMAS), Université de Lorraine, Metz 57073, France

**Keywords:** Ni–Mn–Sb intermetallic compounds, martensitic transformation, orientation relationship, variant organization, electron backscatter diffraction (EBSD), crystallography

## Abstract

The mechanical and magnetic properties of Ni–Mn–Sb intermetallic compounds are closely related to the martensitic transformation and martensite variant organization. However, studies of these issues are very limited. Thus, a thorough crystallographic investigation of the martensitic transformation orientation relationship (OR), the transformation deformation and their impact on the variant organization of an Ni_50_Mn_38_Sb_12_ alloy using scanning electron microscopy/electron backscatter diffraction (SEM/EBSD) was conducted in this work. It is shown that the martensite variants are hierarchically organized into plates, each possessing four distinct twin-related variants, and the plates into plate colonies, each containing four distinct plates delimited by compatible and incompatible plate interfaces. Such a characteristic organization is produced by the martensitic transformation. It is revealed that the transformation obeys the Pitsch relation ({0}_A_ // {2}_M_ and 〈01〉_A_ // 〈2〉_M_; the subscripts A and M refer to austenite and martensite, respectively). The type I twinning plane *K*_1_ of the intra-plate variants and the compatible plate interface plane correspond to the respective orientation relationship planes {0}_A_ and {0}_A_ of austenite. The three {0}_A_ planes possessed by each pair of compatible plates, one corresponding to the compatible plate interface and the other two to the variants in the two plates, are interrelated by 60° and belong to a single 〈11〉_A_ axis zone. The {0}_A_ planes representing the two pairs of compatible plates in each plate colony belong to two 〈11〉_A_ axis zones having one {0}_A_ plane in common. This common plane defines the compatible plate interfaces of the two pairs of plates. The transformation strains to form the variants in the compatible plates are compatible and demonstrate an edge-to-edge character. Thus, such plates should nucleate and grow simultaneously. On the other hand, the strains to form the variants in the incompatible plates are incompatible, so they nucleate and grow separately until they meet during the transformation. The results of the present work provide comprehensive information on the martensitic transformation of Ni–Mn–Sb intermetallic compounds and its impact on martensite variant organization.

## Abstract 2

The martensitic transformation orientation relationship (OR), the transformation deformation and their impact on variant organization in an Ni_50_Mn_38_Sb_12_ alloy have been thoroughly studied by scanning electron microscopy/electron backscatter diffraction (SEM/EBSD), as they are decisive factors for mechanical and magnetic properties. The transformation OR has been identified and the consequent hierarchical organization of martensite variants has been fully revealed.

## 1. Introduction   

The martensitic transformation is a diffusionless solid-state phase transition occurring in alloys, mainly steels, and intermetallic compounds. During the transformation, the structural change from the parent phase to the product phase is realised by a coordinated lattice deformation. To ensure minimum energy consumption, the resultant martensite is usually self-accommodated in terms of transformation strain and possesses invariant habit planes with the parent austenite. Owing to the fact that the properties of alloys can be optimized by martensitic transformation through microstructure modification, the martensitic transformation is still a topic of intensive experimental and theoretical studies. Investigations have been conducted mainly on transformation microstructure characteristics (Maresca *et al.*, 2016 ▸; Wang *et al.*, 2016 ▸; Zhang, Ruimi *et al.*, 2016 ▸), phase transition features (Hilkhuijsen *et al.*, 2013 ▸; De Knijf *et al.*, 2014 ▸; Zolotorevsky *et al.*, 2015 ▸) and property optimization (Zhen *et al.*, 2015 ▸; Findley *et al.*, 2017 ▸).

Other than the various steels, many intermetallic compounds, such as shape-memory materials (NiTi, and Cu-based and Fe-based alloys), also exhibit a martensitic transformation. This transformation is reversible in shape-memory alloys, allowing a recovery of the shape change induced by the forward transformation. Recently, it has been revealed that the newly developed magnetic Ni–Mn–*X* (*X* = Ga, In, Sn, Sb) Heusler-type intermetallic compounds exhibit a martensitic transformation. Moreover, the structure transformation is always accompanied by a magnetic transition, giving rise to a variety of novel magnetic field-driven phenomena. Many studies have been conducted on the exploration of new properties and the characterization of microstructures resulting from the martensitic transformation (Sozinov *et al.*, 2002 ▸; Khovailo *et al.*, 2003 ▸; Sutou *et al.*, 2004 ▸; Krenke *et al.*, 2005 ▸; Pasquale *et al.*, 2005 ▸; Kainuma, Imano, Ito, Morito *et al.*, 2006 ▸; Kainuma, Imano, Ito, Sutou *et al.*, 2006 ▸; Cong *et al.*, 2007 ▸; Khan *et al.*, 2007 ▸; Krenke *et al.*, 2007 ▸; Babita *et al.*, 2009 ▸; Monroe *et al.*, 2012 ▸; Yu *et al.*, 2014 ▸; Huang *et al.*, 2015 ▸; Yang *et al.*, 2015 ▸; Sharma *et al.*, 2016*a* ▸,*b* ▸).

As a new member of this family, the Ni–Mn–Sb system has demonstrated multiple functionalities under an actuating magnetic field, such as the shape-memory effect (SME) (Yu *et al.*, 2014 ▸), the magnetocaloric effect (MCE) (Nayak *et al.*, 2009 ▸; Akkera *et al.*, 2015 ▸; Barman & Kaur, 2015 ▸), giant magnetoresistance (GMR) (Khan *et al.*, 2013 ▸; Sahoo *et al.*, 2013 ▸) and exchange bias (EB) (Sharma Akkera *et al.*, 2013 ▸; Lee *et al.*, 2013 ▸; Barman *et al.*, 2014 ▸). These materials have thus been considered as potential candidates for magnetic shape-memory, magnetic refrigerant and magnetic recording applications. It has been shown that, for the Ni–Mn–Sb system, the occurrence of SME, MCE, GMR and EB is closely related to the martensitic transformation. It has been proved that, by modifying the transformation process, the properties can be considerably improved. For example, Nayak *et al.* (2009 ▸) applied a 1.1 kbar (1 bar = 100 000 Pa) pressure to a polycrystalline Ni_45_Co_5_Mn_38_Sb_12_ alloy during the forward and reverse martensitic transformation processes and obtained a significant increase in entropy from 41.4 to 46 J kg^−1^ K^−1^. Such an enhancement was speculated to be the result of a reorientation of the martensite variants from the original as-transformed microstructure of martensite, driven by the external pressure. However, information concerning the variant organization in the as-transformed microstructure, crystallographic features of the martensitic transformation and their impact on variant organization is still missing. This information is essential for a deep understanding of the specific functionality of these materials and hence serves as a prerequisite for further development of these materials towards practical applications.

In the present work, a thorough experimental examination and crystallographic analysis was conducted on the organization characteristics of the resultant martensite variants, the orientation relationship (OR) of the martensitic transformation and the transformation strain characteristics of an Ni–Mn–Sb alloy, with the aim of working out the underlying mechanisms of the specific variant organization features. High-resolution scanning electron microscopy (SEM) electron backscatter diffraction (EBSD) characterization was utilized to acquire the spatially correlated microstructural and crystallographic orientation information of the martensite variants. The formation mechanism of the martensite microstructure was investigated by analysing the transformation strain compatibility of adjacent martensite variants. This study is expected to provide comprehensive fundamental crystallographic and microstructural information on Ni–Mn–Sb intermetallic compounds for further studies.

## 2. Materials and experimental details   

A polycrystalline Ni–Mn–Sb alloy with the nominal composition Ni_50_Mn_38_Sb_12_ (at.%) was used in the present work. The alloy was prepared by arc melting high-purity elements Ni (99.99 wt%), Mn (99.8 wt%) and Sb (99.995 wt%). An extra 2% (wt%) Mn and an extra 2% (wt%) Sb were added to compensate for the weight loss due to the excessive volatilization of these two elements. The ingot was re-melted four times to ensure a homogeneous composition and then spray cast into copper moulds to obtain dense bulk cylindrical samples. The samples were annealed at 1173 K for 24 h followed by water quenching for compositional homogeneity. The arc melting, spray casting and heat treatment were carried out under an argon atmosphere. The actual chemical composition of the prepared alloy analysed by energy dispersive spectrometry is Ni_49.43_Mn_38.07_Sb_12.50_ (at.%), very close to the nominal composition. The martensitic transformation start and finish temperatures, and the austenitic transformation start and finish temperatures, of the present alloy are 314.94 and 308.85 K, and 328.17 and 334.05 K, respectively (Zhang, Yan *et al.*, 2016 ▸). This indicates that at room temperature the alloy is in the martensite state.

To investigate the microstructure of the martensite and the crystallographic characteristics of the martensitic transformation, parallelepiped samples with sizes of 4 × 6 × 10 mm were cut out of the annealed cylindrical samples by electrical discharge wire-cutting. The samples were mechanically ground with SiC papers and electrolytically polished using a solution of 20% nitric acid and 80% methanol (*v*/*v*). The electrolytic polishing was conducted at 273 K under a continuous multistep mode: 8 V for 10 s, 15 V for 5 s and 18 V for 3 s.

The microstructural and crystallographic characterizations were performed using a field emission gun SEM (JEOL 6500F) equipped with an EBSD acquisition camera (Oxford) and the *Aztec* online acquisition software (Oxford Instruments). Both manual and automatic modes were used for orientation acquisition. Crystallographic calculations, *e.g.* pole figures, misorientation, trace analysis and coordinate transformation, were applied in the crystallographic analyses.

## 3. Results   

### 3.1. Morphological features of 4*O* martensite   

**Figure 1:**
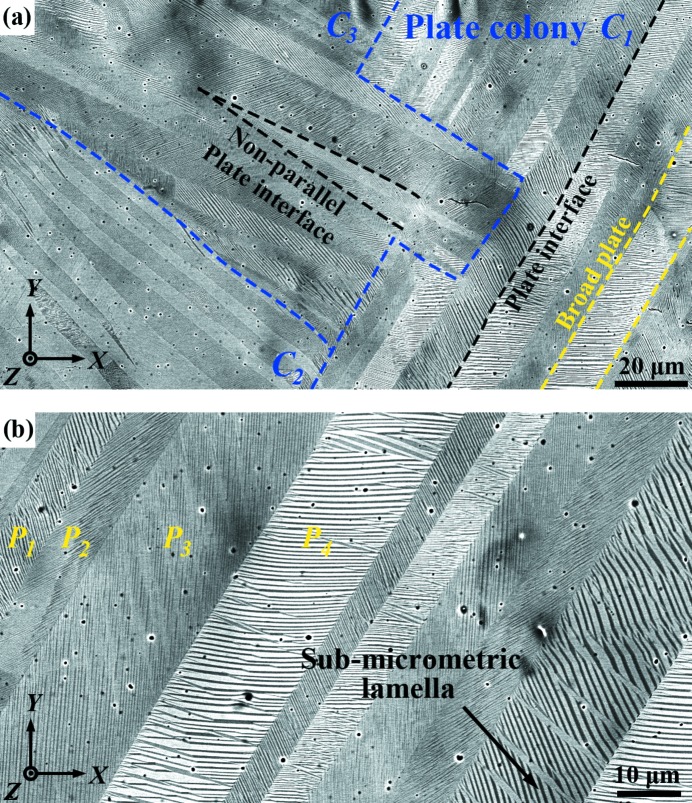
(*a*) A typical backscattered electron (BSE) image of Ni_50_Mn_38_Sb_12_ intragranular martensite. (*b*) A magnified BSE image of the plate colony *C*_1_ in panel (*a*).

At room temperature, the present alloy is composed of 4*O* modulated martensite. Fig. 1 ▸ shows a typical backscattered electron (BSE) micrograph of the martensite in Ni_50_Mn_38_Sb_12_. It is seen that the martensite is organized hierarchically from sub-micrometric lamellae to micrometric plates (outlined by the yellow dashed lines in Fig. 1 ▸ *a*) and from plates into plate groups (outlined by the blue dashed lines in Fig. 1 ▸ *a*). Within each plate, the fine lamellae stretch roughly in the same direction, and within each plate group the plates also stretch in almost the same direction and share an almost straight plate interface, as marked by the black dashed line in Fig. 1 ▸(*a*). For easy reference, we denote the groups of almost-parallel plates a ‘plate colony’ and the plate of lamellae a ‘variant colony’. It can be seen that, in some plate colonies (for example *C*_1_ in Fig. 1 ▸ *a*), the traces of the plate interfaces are parallel, whereas in others (for example *C*_2_ and *C*_3_), the traces are not exactly parallel but deviate one from another by a maximum of 5°, as highlighted in Fig. 1 ▸(*a*). Fig. 1 ▸(*b*) displays the magnified BSE image of one plate colony (*C*_1_) of Fig. 1 ▸(*a*). It is seen that, within the plate colony, the orientation of the lamellar interface trace changes from plate to plate. Each plate colony is composed of four distinct plates in terms of lamellar interface trace orientation, indicated by *P*_1_–*P*_4_ in Fig. 1 ▸(*b*).

### 3.2. Crystallographic correlations between martensite variants   

#### 3.2.1. Intra-plate variants   

**Figure 2:**
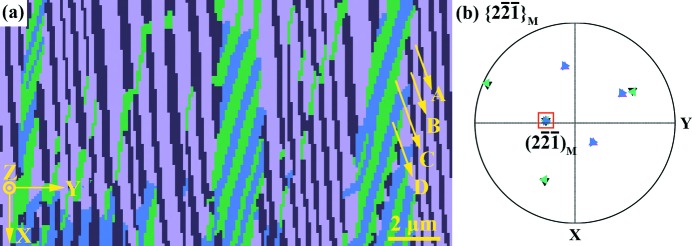
(*a*) Electron backscatter diffraction (EBSD) orientation micrograph of the sub-micrometric lamellar martensite variants in one plate. (*b*) A {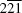}_M_ pole figure of the four variants. The common poles are included in the red square and the poles are presented with the same colours as in panel (*a*).

Further EBSD orientation analysis demonstrated that, within each plate, there are four distinct orientation variants, *A*, *B*, *C* and *D*, as shown in Fig. 2 ▸. The four lamellar or intra-plate variants are related to one another by three kinds of twin relationship [type I twin (*A*/*C* and *B*/*D*), type II twin (*A*/*B* and *C*/*D*) and compound twin (*A*/*D* and *B*/*C*)], as fully determined in our previous work (Zhang, Yan *et al.*, 2016 ▸). Moreover, the four intra-plate variants share one common {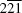}_M_ plane, as shown by the {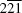}_M_ pole figure in Fig. 2 ▸(*b*). This plane is also their type I twinning plane *K*_1_. Close observation revealed that only type I and type II twin-related variants form the plate interfaces and type II twins appear in a majority. Compound twin-related variants occur only within plates.

#### 3.2.2. Inter-plate variants   

Using the measured orientations of the variants in the four distinct plates in Fig. 1 ▸(*b*) (*P*_1_, *P*_2_, *P*_3_ and *P*_4_), the ORs between adjacent variants in neighbouring plates (connected by the plate interfaces *P*_1_/*P*_2_, *P*_2_/*P*_3_ and *P*_3_/*P*_4_) were analysed. As seen in Fig. 1 ▸(*b*), each variant in one plate can have four possible combinations with the four variants in the other plates. Hence, we calculated the mis­orientation (ω, **d**) and the plane normal to the rotation axis **d** of all the possible variant pairs from plates *P*_1_ to *P*_4_. The results show that, for any variant in plate *P_i_* (*i* = 1, 2, 3 and 4), there exists only one variant in the adjacent plate *P*_*i*+1_ (for *i* = 4, the adjacent plate is *P*_1_) that has a 180° rotation relationship with it. The misorientations of such variant pairs are equivalent at all plate interfaces *P_i_*/*P*_*i*+1_. Table 1 ▸ displays the results for the variant pairs at *P*_1_/*P*_2_ and *P*_2_/*P*_3_. For easy notation, we denote the four distinct variants in plate *P_i_* variants *A_i_*, *B_i_*, *C_i_* and *D_i_*.

**Figure 3:**
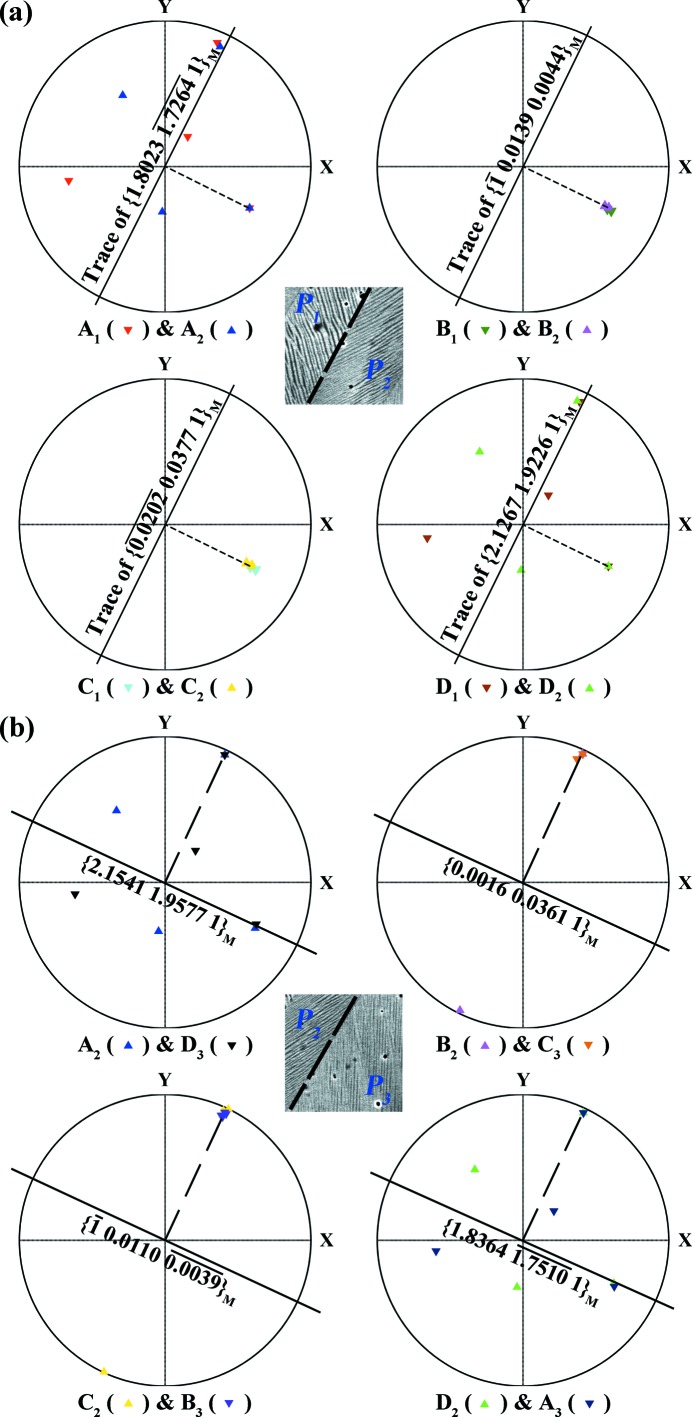
Stereographic projections of the common plane normal to the 180° rotation axis of each interplate variant pair at (*a*) *P*_1_/*P*_2_ and (*b*) *P*_2_/*P*_3_ that possesses a 180° rotation. The trace of the common plane is indicated by a solid black line and the rotation axis by a dashed black line. For comparison, the microstructures with the corresponding plate interface traces are displayed as insets.

We then studied the orientation character of the plane that is normal to the 180° rotation axis of each variant pair. Such a plane should be shared by the corresponding pair of variants. We found that, although the Miller indices of the plane change from pair to pair, the spatial orientations of these planes are very close. For the variant pairs at *P*_1_/*P*_2_ and *P*_3_/*P*_4_, the orientations of these planes coincide with those of the plate interfaces *P*_1_/*P*_2_ and *P*_3_/*P*_4_, as shown by the example *P*_1_/*P*_2_ in Fig. 3 ▸(*a*). In the figure, these planes are represented by their stereographic projections in the macroscopic sample coordinate system, and their traces, as well as the *P*_1_/*P*_2_ plate interface trace, are indicated by black solid lines. The deviation between these planes and the plate interface should be attributed to the experimental imprecision arising from the tilt of the sample for the EBSD measurement. This result indicates that the plate interface should be the mirror plane of the two variants that possess a 180° rotation at *P*_1_/*P*_2_ and *P*_3_/*P*_4_. However, for the variant pairs at *P*_2_/*P*_3_ and *P*_4_/*P*_1_, the orientations of the common planes are not coincident with those of the plate interfaces *P*_2_/*P*_3_ and *P*_4_/*P*_1_ but 90° away, as shown in Fig. 3 ▸(*b*). These inter-plate variant characteristics are confirmed to be the same for the other plate groups. Such characteristic variant organization features suggest that, during the martensitic transformation, *P*_1_–*P*_2_ or *P*_3_–*P*_4_ may form coordinately and grow coordinately. Plate interfaces *P*_2_/*P*_3_ or *P*_4_/*P*_1_ may form when the corresponding plates meet during the transformation. We denote the former plate interfaces ‘compatible interfaces’ and the latter plate interfaces ‘incompatible interfaces’. Knowledge of the transformation ORs should be useful and allows further analysis of the organization features of the present martensite.

### 3.3. Determination of martensitic transformation OR   

#### 3.3.1. Crystal structure and structure simplification   

**Figure 4:**
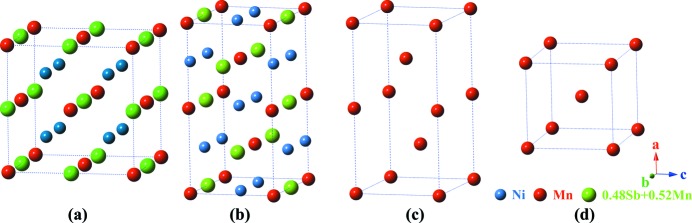
The crystal structures of (*a*) cubic austenite, (*b*) 4*O* modulated martensite and (*c*) simplified 4*O* modulated martensite. (*d*) The average unit cell of the 4*O* modulated martensite.

As specified by our previous work, the martensite of Ni_50_Mn_38_Sb_12_ (used in the present work) possesses a 4*O* (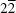) modulated structure of space group *Pmma* (No. 051) with lattice parameters *a*_M_ = 8.5788 Å, *b*_M_ = 5.6443 Å and *c*_M_ = 4.3479 Å (Zhang, Yan *et al.*, 2016 ▸). The austenite has a cubic *L*2_1_ structure in space group 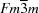 (No. 225) with lattice parameter *a*_A_ = 5.964 Å (Feng *et al.*, 2010 ▸). The subscript ‘A’ indicates the cubic lattice of austenite. According to the published atom occupation information for the austenite (Brown *et al.*, 2010 ▸) (for a very similar composition, Ni_50_Mn_37_Sb_13_) and the structural information for the martensite (Zhang, Yan *et al.*, 2016 ▸), the unit cells of the austenite and martensite can be obtained and are shown in Figs. 4 ▸(*a*) and 4 ▸(*b*). For the sake of simplicity and clarity, the martensite structure is illustrated with only the Mn atoms in Fig. 4 ▸(*c*). By ignoring the structural modulations along the *c* axis of the two Mn atoms at the body centres in each sub-cell, the structure can be further simplified and represented as one sub-cell, as shown in Fig. 4 ▸(*d*). We denote such a cell the average unit cell. The lattice parameters of this cell are 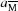 = 4.2894 Å, 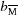 = 5.6443 Å and 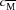 = 4.3479 Å. The subscript ‘
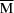
’ indicates the orthorhombic lattice of the average unit cell of the martensite.

#### 3.3.2. Determination of transformation OR by crystallographic calculations   

Generally, the transformation OR is defined by one pair of parallel crystalline planes and one pair of in-plane parallel directions from the corresponding parent and product phases. By consulting the literature, four representative ORs, namely the Bain (Bain & Dunkirk, 1924 ▸), the Kurdjumov–Sachs (K–S) (Kurdjumow & Sachs, 1930 ▸), the Nishiyama–Wassermann (N–W) (Nishiyama, 1934 ▸; Wassermann & Mitt, 1935 ▸) and the Pitsch (Pitsch, 1962 ▸) ORs, were selected as possible transformation ORs for the present alloy. By adapting the Miller indices of the published ORs to the structures of the present austenite and the average cell of the martensite, the plane and direction parallelisms defined by these ORs are as specified in Table 2 ▸.

The above microstructural observations of Ni_50_Mn_38_Sb_12_ reveal that, at room temperature, the martensitic transformation is complete. All the parent austenite has transformed to the product martensite without any retained austenite. Thus, the OR is determined indirectly by inspecting the calculated orientations of the austenite from the measured orientations of the martensite variants originating from the same initial austenite grain under the four ORs listed in Table 2 ▸. The OR that ensures a common austenite orientation should be the effective one. Here we used the orientations of the four variants (*A*, *B*, *C* and *D*) displayed in Fig. 2 ▸(*a*) to determine the effective transformation OR. To ensure the accuracy of the determination, we used the mean orientation of the four martensite variants.

**Figure 5:**
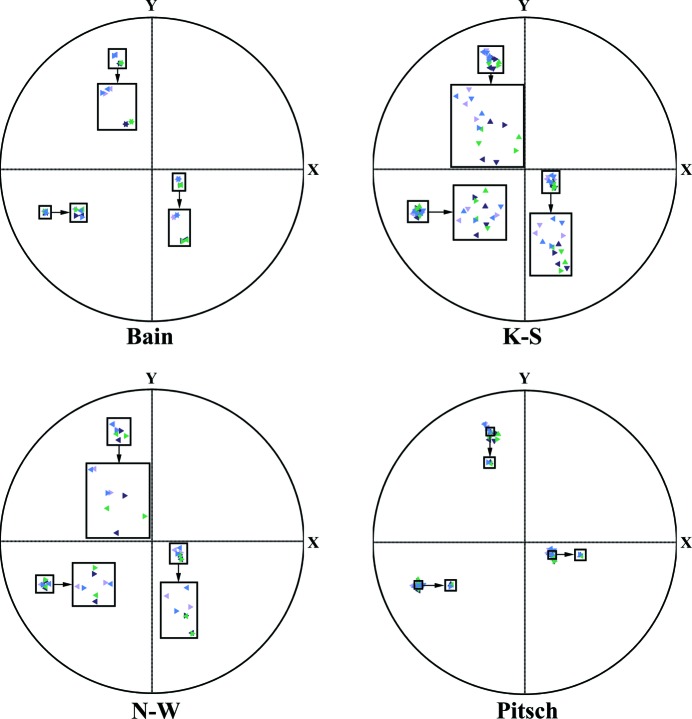
{001}_A_ stereographic projections of austenite under the four ORs. Orientations obtained from different martensite variants are distinguished with different colours that are consistent with those of the four variants in Fig. 2 ▸(*a*): mauve for variant *A*, dark blue for variant *B*, green for variant *C* and blue for variant *D*. The non-equivalent austenite orientations obtained from one martensite variant are differentiated by the orientations of the triangular symbols. The clusters of poles in each stereographic projection are further magnified to give a convenient visualization of the positions of the poles.

With the mean orientation of the four variants, the rotation matrix 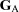 that expresses the orientation of the possible original austenite with respect to the sample coordinate system can be calculated *via* the assumed OR using the following equation:
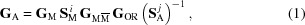
where **G**_M_ is the rotation matrix representing the orientation of the martensite variant with respect to the sample coordinate system, 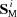 (*i* = 1–4) and 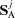 (*j* = 1–24) are the corresponding rotational symmetry matrices of the orthorhombic and cubic crystal systems, respectively, 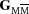 is the coordinate transformation matrix from the orthnormal crystal coordinate frame set to the 4*O* modulated martensite to the orthnormal crystal coordinate frame set to the average unit cell of martensite, and **G**_OR_ is the coordinate transformation matrix from the orthonormal crystal coordinate frame set to the average unit cell of martensite to the cubic coordinate system set to the lattice basis of austenite under a given OR listed in Table 2 ▸. The calculated orientations of the austenite are represented with their {001}_A_ stereographic projections in the sample coordinate system and shown in Fig. 5 ▸. Due to the symmetry of the cubic system, one austenite orientation is represented with three distinct but equivalent {001}_A_ poles that are marked with triangles of the same colour and orientation in the figures. The colours of the triangles are consistent with those of the martensite variants displayed in Fig. 2 ▸(*a*). Due to crystal symmetry, one measured martensite variant can generate several distinct austenite orientations depending on the OR. If the OR is effective for the transformation of the present alloy, the orientations of the austenite calculated from the four martensite variants should share a common austenite orientation. That means that each of the three {001}_A_ poles from the corresponding variants should superimpose on the {001}_A_ stereographic projection. It can be seen from Fig. 5 ▸ that, among all the selected ORs, only the Pitsch relation ensures a common austenite orientation from all the martensite variants, indicating that this OR could be the effective one. To quantify further the mismatch between the closest orientations of austenite calculated from the variants under the four ORs, the disorientation angles between each pair of austenite orientations were calculated and these are listed in Table 3 ▸. Obviously, under the Pitsch OR the disorientation angles are the smallest, which confirms that this OR, specified as {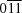}_A_ // {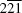}_M_ and 〈
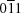
〉_A_ // 〈
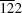
〉_M_, is the effective OR for the transformation from the austenite to the 4*O* modulated martensite.

### 3.4. Impact of transformation strain on variant organization   

#### 3.4.1. Crystallographic correlation between austenite and martensite variants   

With the determined transformation OR, the crystallographic correlation between the two phases can be studied further. Since the parent austenite and the product 4*O* modulated martensite possess the parallel relationship {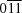}_A_ // {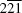}_M_ and 〈
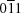
〉_A_ // 〈
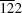
〉_M_ under the Pitsch relation, each {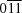}_A_ plane can provide four coplanar {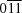}_A_ – 〈
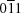
〉_A_ combinations by reversing the sign of the {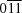}_A_ plane and that of the 〈
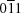
〉_A_ direction, as shown in Table 4 ▸, thus giving rise to four distinct martensite variants. As the {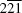}_M_ plane of the four variants that result from the common {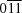}_A_ plane are parallel, these four variants are, in fact, those belonging to one variant plate, as revealed experimentally above (Fig. 2 ▸ *b*).

**Figure 6:**
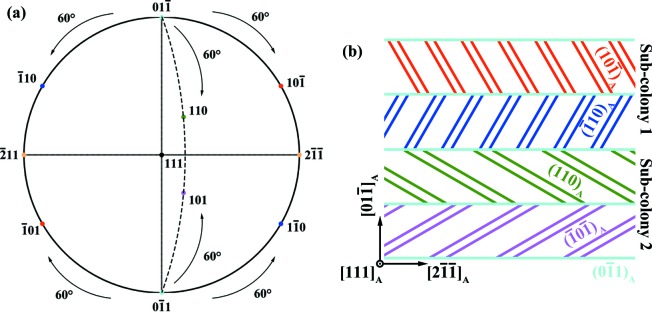
(*a*) (111)_A_ standard stereographic projection. The (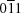)_A_, (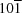)_A_ and (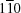)_A_ planes that are located on the circumference belong to the [111]_A_ axis zone, whereas the (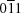)_A_, (110)_A_ and (101)_A_ planes that are located on the dashed arc line belong to the [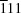]_A_ axis zone. (*b*) A microstructural schema of the corresponding plate colony containing two pairs of compatible plates, one being related to the (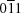)_A_, (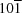)_A_ and (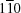)_A_ group and the other to the (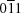)_A_, (110)_A_ and (101)_A_ group.

With the determined OR between the austenite and the martensite, the interface between plates in each plate colony can be correlated with the planes of the austenite. As revealed by the above experimental results, the compatible plate interface is defined by the plane that is normal to the 180° rotation axis (listed in Table 1 ▸) shared by the pairs of martensite variants from neighbouring plates. The corresponding austenite plane can thus be determined. Calculation showed that it is one of the {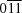}_A_ planes (with a very small angular deviation of about 0.75°). In fact, the three {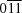}_A_ planes, one corresponding to the plate interface and the other two to the common {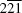}_M_ planes of the martensite variants (four in each plate) in adjacent plates, are related by 60°. In other words, the three {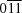}_A_ planes belong to one 〈
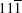
〉_A_ axis zone. Due to the cubic symmetry, one {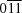}_A_ plane belongs to two 〈
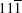
〉_A_ axis zones and is related to the other four {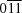}_A_ planes in the two zones by 60°. For example (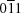)_A_, (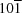)_A_ and (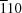)_A_ in the [111]_A_ axis zone, and (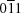)_A_, (110)_A_ and (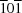)_A_ in [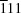]_A_, as shown in Fig. 6 ▸(*a*). If we take the (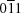)_A_ plane, *i.e.* the common plane in the two groups, as the compatible plate interface, two distinct compatible plate pairs can be constructed, as illustrated in Fig. 6 ▸(*b*). Each plate group contains two distinct compatible plate pairs and these two pairs correspond to the four distinct plates in one plate colony.

This result is completely consistent with the observed microstructure features. Such a specific variant selection rule in a plate colony should be related to the transformation strain characteristics of the constituent variants and their interplay. Thus, analysis of the transformation strain is imperative to figure out the underlying mechanisms.

#### 3.4.2. Transformation strain compatibility at plate interfaces in plate colony   

With the determined transformation OR, the structure deformation to form the martensite variants at the two kinds of plate interface within one plate colony were further calculated using the phenomenological theory of martensitic transformation (Wechsler *et al.*, 1953 ▸; Bowles & Mackenzie, 1954 ▸; Ball & James, 1987 ▸; Jin & Weng, 2002 ▸; Bhattacharya, 2003 ▸; Balandraud *et al.*, 2010 ▸) to examine their geometric compatibility at the two kinds of plate interface (compatible and incompatible). Here we take the variants in plates *P*_1_, *P*_2_, *P*_3_ and *P*_4_ in Fig. 1 ▸(*b*) for the compatibility analysis.

**Figure 7:**
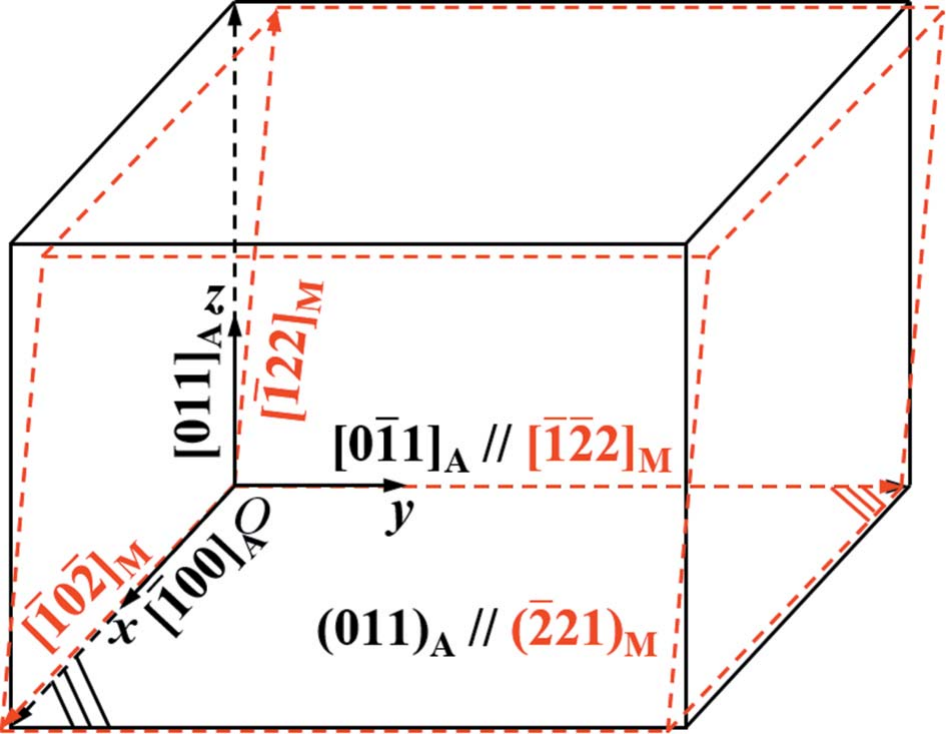
Lattice correspondences between the parent austenite (black) and the product martensite (red) under the Pitsch OR.

The deformation gradient tensor to describe the structure transformation from austenite to each corresponding martensite variant was established by examining the atomic correspondences of the original austenite and the variants under the Pitsch OR, as illustrated in Fig. 7 ▸. The deformation gradient tensor ***F***_or_ in the OR frame (*xyz*), can be constructed as follows:
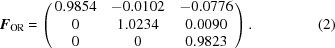
It can be further expressed in the Bravais lattice basis of austenite by a coordinate transformation
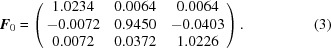
Thus the deformation gradient tensors of the 24 theoretical variants can be calculated using the following equation:
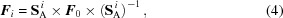
for *i* = 1–24, where 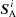 are the rotational symmetry elements of the cubic crystal system. Then, by examining the measured orientation of the 16 variants *A_i_*, *B_i_*, *C_i_* and *D_i_* (*i* = 1, 2, 3 and 4) in plates *P*_1_–*P*_4_ in Fig. 1 ▸(*b*), we can obtain the deformation gradient tensors of the 16 variants in the four plates expressed in the Bravais lattice basis of austenite as listed in Table 5 ▸.

According to the phenomenological theory of martensitic transformation, the transformation is characterized by an invariant plane strain. In mathematics, if such a condition is achieved, one of the eigenstrains of the transformation deformation should be equal to 1. In reality, this means the transformation can produce an invariant interface between austenite and martensite, the so-called habit plane. For the present alloy, the eigenstrains of each single variant are 0.9458, 1.0165 and 1.0303, respectively. None of them equals 1. That means that, by forming a single martensite variant, the invariant plane strain condition cannot be satisfied. Thus locally, two twin-related variants are needed and a sandwich-structured variant agglomeration is usually formed to achieve an invariant habit plane. This corresponds exactly to what we observed in the microstructure. Thus, the total deformation gradient tensor of the paired variants ***F*** can be described by the following equation (Jin & Weng, 2002 ▸):
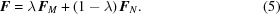
Here, ***F***_*M*_ and ***F***_*N*_ are the deformation gradient tensors of the two constituent variants *M* and *N*, and λ is the volume fraction of variant *M*, enabling an invariant habit interface. The results of these calculations show that both the type I twin combination and the type II twin pair can form invariant habit planes. The volume fractions of the two pairs are 0.7201:0.2799 and 0.7179:0.2821, respectively, which are in good accordance with our experimental results.

**Figure 8:**
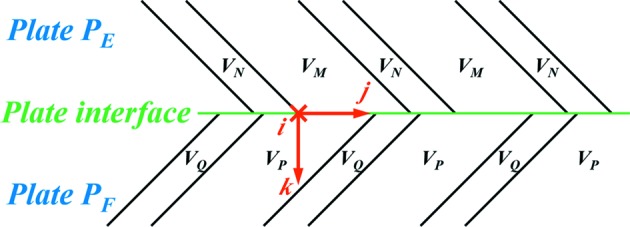
Illustration of the variant composition in the vicinity of the plate interface based on the morphological features described in Section 3.1 and Section 3.2. The *ijk* coordinate frame is orthonormal, with *i* parallel to the intersection line of the twinning plane of variant pair *V_P_* and *V_Q_* and the plate interface, *k* normal to the plate interface, and *j* the vector cross product of *k* and *i*.

With the above results, we further studied the transformation strain characteristics of different variant pairs in one plate colony. Here we take variant pairs *A*_1_/*B*_1_, *A*_2_/*B*_2_, *C*_3_/*D*_3_ and *C*_4_/*D*_4_ in plates *P*_1_, *P*_2_, *P*_3_ and *P*_4_ in Fig. 1 ▸(*b*) as examples. Variant pairs *A*_1_/*B*_1_ and *A*_2_/*B*_2_ (*C*_3_/*D*_3_ and *C*_4_/*D*_4_) form the compatible plate interface, and *A*_1_/*B*_1_ and *C*_3_/*D*_3_ (*A*_2_/*B*_2_ and *C*_4_/*D*_4_) the incompatible interface. The plate interface in the plate colony of Fig. 1 ▸(*b*) corresponds to (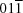)_A_. The deformation gradient tensors of the four variant pairs 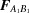, 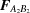, 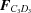 and 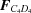 are calculated and expressed in the *ijk* coordinate frame, as illustrated in Fig. 8 ▸, where the *i* axis is parallel to the intersection of the twinning plane of variant pair *A*_1_/*B*_1_ on the plate interface (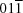)_A_, the *k* axis is normal to the plate interface (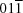)_A_ and the *j* axis is the vector cross product of the two axes *k* and *i*: 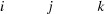


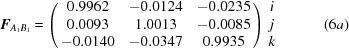


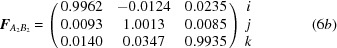


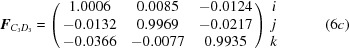


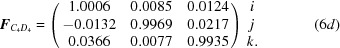
In the deformation gradient tensor, the three diagonal elements ***F***(*i*, *i*), ***F***(*j*, *j*) or ***F***(*k*, *k*) represent normal contraction (<1) or elongation (>1) along the *i*, *j* or *k* axis, whereas the six off-diagonal elements represent shears [for example ***F***(*i*, *j*) represents a shear in the ***i*** direction on the plane normal to ***j***]. For variant pairs *A*_1_/*B*_1_ and *A*_2_/*B*_2_ (*C*_3_/*D*_3_ and *C*_4_/*D*_4_) connected by a compatible plate interface, the components of the two tensors 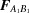 and 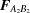 (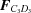 and 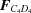) demonstrate a matched edge-to-edge deformation character. All the corresponding elements in the two tensors have exactly the same absolute value with the sign of the shear components ***F***(*i*, *k*), ***F***(*j*, *k*), ***F***(*k*, *i*) and ***F***(*k*, *j*) reversed. From Fig. 8 ▸, we can see that, under such a deformation manner, the two variant colonies keep a matched transformation deformation along the directions of ***i*** and ***j*** [the same ***F***(*j*, *i*) and ***F***(*i*, *j*)]. For the shear deformation either in the direction of ***k*** or on the plane normal to ***k***, the sign of the strain is reversed. This keeps the deformation on the two sides of the plate interface symmetrical and balanced. Thus the transformation strain at the interface is totally compatible, ensuring a coordinated and compatible growth of the martensite variants in the two plates.

For variant pairs *A*_2_/*B*_2_ and *C*_3_/*D*_3_ (*A*_1_/*B*_1_ and *C*_4_/*D*_4_) that are connected by the incompatible plate interface, only the ***F***(*k*, *k*) element has the same value whereas the others do not, suggesting that the plate interface should be formed when the two plates meet during the transformation process. In such a case, the normal strain ***F***(*k*, *k*) and the two shear strains ***F***(*k*, *i*) and ***F***(*k*, *j*) in the **k** direction affect the orientation of the plate interface. As the two shear strains ***F***(*k*, *i*) and ***F***(*k*, *j*) from the two pairs of variants from the two plates are not totally symmetric, the orientation of the incompatible plate interfaces could deviate from that of the compatible plate interfaces. As the two shear strains are relatively small, the deviation should be small. Depending on the orientation of these interfaces with respect to the observing plane, the traces of the in­compatible plate interfaces could be parallel to those of the compatible ones, as in the case of the colony in Fig. 1 ▸(*b*), or at certain angle, as in the case of plate colonies *C*_2_ and *C*_3_ in Fig. 1 ▸(*a*).

According to the symmetry of the cubic crystal system, one can have six equivalent {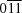}_A_ planes, each of which can be associated with one plate colony. Thus in total, one original austenite grain can produce six distinct plate colonies. Our experimental observations revealed only three distinct plate colonies. As our microstructure observations were conducted on a two-dimensional sample section, it is possible that there are six distinct plate colonies in the three-dimensional space of austenite grains. To verify this hypothesis, we studied the overall transformation strain of the plate colonies to see if there is any request for strain accommodation between colonies. We can find, in Fig. 1 ▸(*b*), that the two distinct plates whose transformation strains are compatible at the plate interface (*P*_1_ and *P*_2_, and *P*_3_ and *P*_4_) always possess the same width. Plate *P*_3_ (and *P*_4_) is about thrice as wide as plate *P*_1_ (and *P*_2_). Hence, we calculate the total deformation gradient of these four plates with the four calculated tensors of variant pairs *A*_1_ *B*_1_, *A*_2_ *B*_2_, *C*_1_ *D*_1_ and *C*_2_ *D*_2_ given in equations (6*a*)–(6*d*) [Disp-formula fd8] [Disp-formula fd9], using the following form: 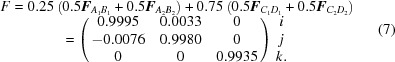
From the tensor in equation (7) one can see that four of the six shear strains are zero. The remaining two shear strains, ***F***(*j*, *i*) and ***F***(*i*, *j*), are also very small compared with those in equations (6*a*)–(6*d*) [Disp-formula fd8] [Disp-formula fd9]. For the three diagonal elements which represent the normal strains, they are very close to 1. This indicates that the transformation gradient of these four plates is very close to the identity matrix representing the original austenite. This means that, within each plate colony, the transformation strain is self-accommodated and there is almost no request for strain accommodation from other colonies. Hence, different plate colonies could form randomly in the original austenite grains. The plate colony interfaces should also form randomly when plate colonies meet during the transformation process. This explains why the plate colony interfaces are irregular, as outlined with the blue dashed lines in Fig. 1 ▸(*b*), and the local transformation strains close to the colony interfaces are not compatible. Therefore, the specific geometric combination of the martensite plate colony should result from the self-accommodation of elastic strains generated by the structural transformation (from austenite to martensite). Hereto, the martensitic transformation OR and the associated hierarchical martensite variant organization features of Ni_50_Mn_38_Sb_12_ are fully detected, which will provide fundamental information for further investigation of property optimization of Ni–Mn–Sb intermetallic materials.

## 4. Summary   

In this work, martensite variant organization features and the underlying formation mechanism in the Ni_50_Mn_38_Sb_12_ intermetallic compound has been thoroughly investigated by SEM/EBSD, the spatially correlated microstructure and crystallographic orientation analysis technique, and crystallographic calculations. The main results are as follows:

(i) The martensite variants are hierarchically organized into plates and the plates into plate colonies. Each plate contains four distinct variants and each plate colony four distinct plates, with a total of 16 distinct variants.

(ii) The plates are separated by two kinds of plate interfaces, compatible and incompatible, depending on whether the interface plane is constituted of the common planes shared by the variants connected by the interface or not.

(iii) The martensitic transformation obeys the Pitsch OR specified as {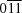}_A_ // {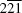}_M_ and 〈
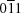
〉_A_ // 〈
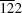
〉_M_. Such an OR results in a specific geometric configuration of the plate colonies. The four variants in each plate share one {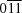}_A_ plane and the compatible plate interface corresponds to another {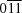}_A_ plane. The three {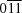}_A_ planes possessed by each pair of compatible plates, one corresponding to the compatible plate interface and the others to the four variants in each plate, are interrelated by 60° and belong to one 〈
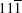
〉_A_ axis zone. The characteristic {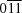}_A_ planes of the two pairs of compatible plates in each plate colony belong to two 〈
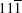
〉_A_ axis zones having one {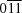}_A_ plane in common. This common plane defines the compatible plate interfaces of the two pairs of plates as well as the plate colony. Hence, in theory, six distinct plate colonies should be produced in one original austenite, even though only three colonies were observed in this work.

(iv) The specific variant organization feature in this Ni–Mn–Sb alloy originates from the specific lattice deformation for the structure transformation. For compatible plates, the transformation strains for the formation of the variants are totally compatible at the plate interface, demonstrating an edge-to-edge character. Thus, compatible plates should form and grow simultaneously. For the incompatible plates, the transformation strains at the plate interface are not compatible, so the interface should be formed when plates meet during the transformation process.

The results of the present work provide basic information for Ni–Mn–Sb intermetallic compounds and could be useful for interpreting their magnetic and mechanical characteristics associated with the martensitic transformation, and for further investigation of martensitic transformation crystallography of these materials.

## Figures and Tables

**Table 1:** 180° rotation (ω, **d**) and the plane normal to the rotation axis **d** of the interplate variant pairs in plates *P*_1_ and *P*_2_, and *P*_2_ and *P*_3_

Plate	Variant pair	Misorientation angle ω (°)	Rotation axis **d** (in the lattice basis of the 4*O* modulated martensite)	Plane normal to **d** (in the lattice basis of the 4*O* modulated martensite)
*P*_1_/*P*_2_	*A*_1_ and *A*_2_	179.7740	〈 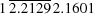 〉_M_	{ 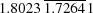 }_M_ (3.47° from { 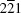 }_M_)
	*B*_1_ and *B*_2_	179.5442	〈 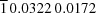 〉_M_	{ 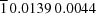 }_M_ (1.31° from { 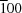 }_M_)
	*C*_1_ and *C*_2_	179.1587	〈 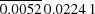 〉_M_	{ 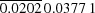 }_M_ (1.76° from {001}_M_)
	*D*_1_ and *D*_2_	179.5321	〈1 2.0884 1.8306〉_M_	{2.1267 1.9226 1}_M_ (2.38° from {221}_M_)
*P*_2_/*P*_3_	*A*_2_ and *D*_3_	179.6586	〈1 2.0995 1.8073〉_M_	{2.1541 1.9577 1}_M_ (2.26° from {221}_M_)
	*B*_2_ and *C*_3_	179.3175	〈0.0004 0.0214 1〉_M_	{0.0016 0.0361 1}_M_ (1.59° from {001}_M_)
	*C*_2_ and *B*_3_	179.1297	〈 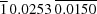 〉_M_	{ 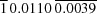 }_M_ (1.05° from { 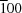 }_M_)
	*D*_2_ and *A*_3_	179.1042	〈 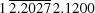 〉_M_	{ 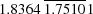 }_M_ (3.12° from { 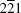 }_M_)

**Table 2:** Plane and direction parallelisms defined by the four ORs adapted to the structure of the austenite and the average structure of the present martensite

OR	Parallel lattice plane and vector in two phases
Bain relation	(010)_A_ // 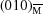 and [001]_A_ // 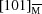
K–S relation	( 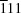 )_A_ // 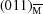 and [ 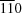 ]_A_ // 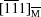
N–W relation	(111)_A_ // 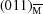 and [ 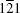 ]_A_ // 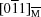
Pitsch relation	( 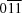 )_A_ // 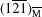 and [ 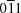 ]_A_ // 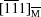

**Table 3:** Disorientation angles between calculated austenite orientations obtained under different ORs

	Disorientation angle for different ORs (°)			
Variant pair	Bain	K–S	N–W	Pitsch
*A* and *B*	4.53	0.07	2.13	0.07
*C* and *D*	4.83	0.40	2.30	0.40
*A* and *C*	4.66	1.86	2.29	0.18
*B* and *D*	4.69	2.06	2.47	0.28
*A* and *D*	0.74	2.12	2.67	0.30
*B* and *C*	0.76	1.80	2.58	0.20

**Table 4:** OR between the original austenite and the four martensite variants *A*, *B*, *C* and *D* shown in Fig. 2 ▸(*a*)

Variant	OR	Variant	OR
*A*	( 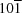 )_A_ // ( 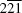 )_M_	*B*	( 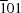 )_A_ // ( 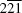 )_M_
	[ 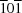 ]_A_ // [ 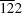 ]_M_		[ 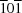 ]_A_ // [ 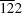 ]_M_
*C*	( 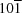 )_A_ // ( 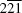 )_M_	*D*	( 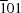 )_A_ // ( 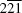 )_M_
	[101]_A_ // [ 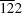 ]_M_		[101]_A_ // [ 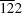 ]_M_

**Table 5:** Deformation gradient tensor ***F*** of variants in plates *P*_1_–*P*_4_ in Fig. 1 ▸(*b*) expressed in the Bravais lattice basis of austenite

Variant	Deformation gradient tensor	Variant	Deformation gradient tensor
*A*_1_	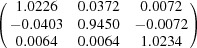	*B*_1_	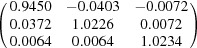
*C*_1_	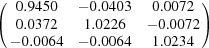	*D*_1_	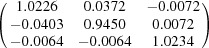
*A*_2_	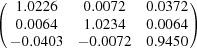	*B*_2_	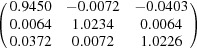
*C*_2_	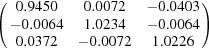	*D*_2_	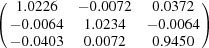
*A*_3_	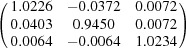	*B*_3_	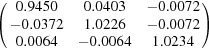
*C*_3_	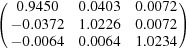	*D*_3_	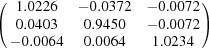
*A*_4_	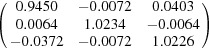	*B*_4_	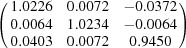
*C*_4_	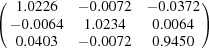	*D*_4_